# Neurocognitive functioning in individuals with congenital central hypoventilation syndrome

**DOI:** 10.1186/s12887-020-2006-5

**Published:** 2020-05-06

**Authors:** Kelly T. Macdonald, Ricardo A. Mosquera, Aravind Yadav, Maria C. Caldas-Vasquez, Hina Emanuel, Kimberly Rennie

**Affiliations:** 1 Department of Psychology, University of Houston, Houston, TX USA; 2 Department of Pediatrics, 2Department of Pediatrics, McGovern Medical School at the University of Texas Health Science Center, Houston, TX USA

**Keywords:** CCHS, Neurocognition, PARMs

## Abstract

**Background:**

Congenital central hypoventilation syndrome (CCHS) is a rare disorder characterized by respiratory system abnormalities, including alveolar hypoventilation and autonomic nervous system dysregulation. CCHS is associated with compromised brain development and neurocognitive functioning. Studies that evaluate cognitive skills in CCHS are limited, and no study has considered cognitive abilities in conjunction with psychosocial and adaptive functioning. Moreover, the roles of pertinent medical variables such as genetic characteristics are also important to consider in the context of neurocognitive functioning.

**Methods:**

Seven participants with CCHS ranging in age from 1 to 20 years underwent neuropsychological evaluations in a clinic setting.

**Results:**

Neurocognitive testing indicated borderline impaired neurocognitive skills, on average, as well as relative weaknesses in working memory. Important strengths, including good coping skills and relatively strong social skills, may serve as protective factors in this population.

**Conclusion:**

CCHS was associated with poor neurocognitive outcomes, especially with some polyalanine repeat expansion mutations (PARMS) genotype. These findings have important implications for individuals with CCHS as well as medical providers for this population.

## Background

Congenital central hypoventilation syndrome (CCHS; OMIM #209880) is a rare disorder with an autosomal dominant mode of inheritance, occurring in 1 in 200,000. CCHS typically presents within the neonatal period and is characterized by respiratory system dysregulation, including alveolar hypoventilation with insensitivity to resultant hypoxemia and hypercarbia [1]. CCHS patients often have autonomic nervous system (ANS) dysregulation, including temperature dysregulation, transient abrupt asystoles, severe breath holding spells, altered gut motility, severe constipation, pupillary abnormalities, and decreased perception of pain [2–4]. The paired-like homeobox 2B (*PHOX2B*) gene was identified as the disease-defining gene for CCHS [5]. Due to potential for repeated hypoxemia and hypercarbia among individuals with CCHS, neurocognitive functioning is often impaired [6], and comprehensive neuropsychological assessment (which includes neurocognitive testing as well as consideration of psychosocial functioning and adaptive skills) has been recommended as part of routine medical care among this population [7]. While a few studies have evaluated neural abnormalities in CCHS patients [8–10], only a handful of studies have examined neurocognitive functioning [11–15]. The limited available data from these studies demonstrates overall intellectual functioning falling within the borderline impaired to low average range but with substantial variability. However, studies that also consider psychosocial outcomes, including emotional and behavioral symptoms as well as adaptive skills, are limited [8, 12]. In order to better inform medical, psychological, and educational interventions for this population, it is important to characterize these aspects of neuropsychological functioning.

It is also important that these outcomes be considered in the context of genetic information (i.e., presence of polyalanine repeat expansion mutations, or PARMs). In their most recent clinical policy statement, the American Thoracic Society [1] summarized findings regarding the role of PARMs and non-PARMs (NPARMs) in CCHS. In addition to the mutation in the *PHOX2B* gene, which is required for a diagnosis, over 90% of individuals with CCHS will also be heterozygous for an inframe PARM coding for 24 to 33 alanines in the mutated protein. Genotypic variations are associated with different disease severity. For example, patients with genotypes from 20/27 to 20/33 typically require continuous ventilatory support.

The purpose of the current study is to describe neuropsychological functioning among individuals with CCHS by considering their cognitive skills in concert with their psychosocial and adaptive outcomes and in the context of a relevant medical variable: presence of PARMS vs. NPARMS. We expect that PARMs will be related to poorer neurocognitive outcomes.

## Method

### Participants

IRB approval was obtained from the institution of the investigators in order to complete a retrospective chart review, and no further permissions were required. We drew the current sample (*N* = 7) of *PHOX2B* confirmed CCHS patients from a comprehensive pediatric care clinic housed in a large medical center in the southwestern United States. The clinic provides comprehensive care for patients with acute and chronic conditions, including children with rare pulmonary conditions. The team includes pediatricians, pediatric Pulmonologists, nurse practitioners, neuropsychologists, and social workers.

Participants ranged in age from 12 months to 20 years (*M* = 7.43 years, SD = 6.55 years), including 5 females and 2 males. Six patients were Hispanic and one was African American. Three of the participants had the 20/25 PARM genotype, one had 20/26 genotype, and three had 20/27 genotype. None of our participants were heterozygous for an NPARM in the *PHOX2B* gene. All three participants with 20/27 genotype required ventilatory support 24 hours per day. The participant with 20/26 genotype also required support. However, there was variability across the three participants with the 20/25 genotype: one required 24 hour/day ventilation, one needed support only at night, and one did not require support.

### Measures

Neuropsychological assessment for CCHS patients occurred in the context of routine medical care, as recommended by the American Thoracic Society [1]. All assessments were administered by a trained graduate student who was supervised by a licensed neuropsychologist. Neurocognitive tests were administered while caregivers completed rating scales of psychosocial and adaptive skills. Due to acute illness, the full testing battery was not administered to one patient.

Neurocognitive functioning, including an estimate of IQ, was obtained with the Wechsler Intelligence Scales for five participants. Three patients were administered the **Wechsler Intelligence Scale for Children, Fifth Edition (WISC-V)** [16], which is an individual test of general intelligence for children aged 6 to 16. One patient was administered the **Wechsler Preschool and Primary Scale of Intelligence, Fourth Edition (WPPSI-IV)** [17], which evaluates cognitive development in children aged 2 to 7 years and one patient was administered the **Wechsler Adult Intelligence Scale, Fourth Edition (WAIS-IV)** [18], which evaluates cognitive functioning among individuals aged 16 to 90 years. The final patient was administered the **Bayley Scales of Infant and Toddler Development, Third Edition (Bayley-III)** [19], which evaluates the developmental functioning of infants and children aged 1–42 months.

The **Behavioral Assessment System for Children, Second Edition (BASC-2)** [20] was used to evaluate psychosocial functioning (i.e., emotional, behavioral symptoms) in six patients. Caregiver ratings of executive functions were obtained with the **Behavioral Rating Inventory of Executive Function (BRIEF)** [21]. Three patients were administered the BRIEF (ages 5 to 18) and two participants were administered the **BRIEF, Preschool Edition (BRIEF-P** [22]; ages 2 to 5). Adaptive functioning was evaluated with the **Adaptive Behavior Assessment Scales, Third Edition (ABAS-3)** [23] for six patients.

### Data analysis

We provide test results for each subject, including subtest scores and composite index scores, as well as means and standard deviations for each score. Due to our small sample size we do not report results from statistical tests, including correlations. Although we computed correlations among primary index scores, results demonstrated spuriously high correlation coefficients that cannot be interpreted meaningfully due to the small sample. Because of the potential for misleading conclusions about the population-level relationships between these scores, we do not report those results here. Unless specifically noted, results discussed below refer to standard scores (*M* = 100, *SD* = 15).

## Results

Demographics, medical variables, and IQ scores are summarized in Table 1.

**Table 1:** Demographics, Medical Variables, and IQ Scores

Participant #	Age	Race/Ethnicity	PARMs	NPARMs	Ventilatory support	FSIQ*
1	1	Hispanic	20/25	None detected	Yes	70^*^
2	2	Hispanic	20/27	None detected	Yes	79
3	4	African American	20/26	None detected	Yes	
4	7	Hispanic	20/25	None detected	Night only	83
5	9	Hispanic	20/27	None detected	Yes	45
6	10	Hispanic	20/25	None detected	No	106
7	20	Hispanic	20/27	None detected	Yes	51
Mean	7.43					72.33
SD	6.55					22.36

*Note:* PARMs = polyalanine repeat expansion mutations; NPARMS = non-polyalanine repeat expansion mutations; FSIQ = Full Scale IQ from the WPPSI-IV, WISC-V, or WAIS-IV

* FSIQ was not available for participant 3. The Cognitive Composite score from the Bayley-III was used for Participant 1 as a proxy for FSIQ

### Relationship between IQ scores and PARMs

On average, intellectual functioning fell in the borderline impaired range (*M =* 72.33, *SD* = 22.36). Participants with the 20/27 genotype had, on average, substantially lower IQ (*M* = 58.33, *SD* = 18.15) than those with the 20/25 genotype (*M* = 86.33, 18.23). Among the three participants with the 20/25 genotype, a clear relationship emerged between need for ventilatory support and IQ, such that 24 hour/day support was associated with IQ in the impaired range, partial support (nighttime only) was associated with low average IQ, and no ventilatory support was associated with average IQ.

Wide variability in our sample’s age range made direct comparison between tests difficult; thus, we separated the tests by domain and summarized the general pattern of results, making direct comparisons where possible. All scores are reported by domain in Tables 2, 3, 4, 5, 6, 7 and 8.

**Table 2:** Verbal Outcomes

Participant #	FSIQ	VCI	Similarities*	Vocabulary*	Receptive Vocabulary*	Information*	Picture Naming*
1	70	77			5		
2	79	71				5	2
3							
4	83	92	10	7			
5	45	59	1	4			
6	106	111	13	11			
7	51	63	3	4		4	
Mean	72.33	78.83	6.75	6.50		4.5	
SD	22.36	19.58	5.68	3.32		0.71	

*Note*: VCI = Verbal Comprehension Index from the Wechsler scales. Similarities and Vocabulary are subtests from the WISC-V and WAIS-IV. Receptive Vocabulary is a subtest from the Bayley-III. Information is a subtest from both the WPPSI-IV and the WAIS-IV. Picture Naming is a subtest from the WPPSI-IV

*Scores for individual subtests are reported as scaled scores, *M* = 10, *SD* = 3

**Table 3:** Nonverbal Outcomes

Participant #	FSIQ	VSI	FRI	Block Design*	Matrix Reasoning*	Figure Weights*	Visual Puzzles*	Object Assembly*
1	70							
2	79	83		6				8
3								
4	83	89	88	10	5	11	6	
5	45	45	58	1	1	4	1	
6	106	111	106	10	10	12	14	
7	51		58	1	3		5	
Mean	72.33	82.00	77.50	5.60	4.75	9.00	6.50	
SD	22.36	27.45	23.69	4.51	3.86	4.36	5.45	

*Note*: VSI = Visual Spatial Index from the WPPSI-IV and the WISC-V. FRI = Fluid Reasoning Index from the WISC-V and WAIS-IV. Block Design is a subtest on all three Wechsler scales. Matrix Reasoning is a subtest on the WISC-V and WAIS-IV. Figure Weights is a subtest from the WISC-V. Visual Puzzles is a subtest from the WISC-V and WAIS-IV. Object Assembly is a subtest from the WPPSI-IV

*Scores for individual subtests are reported as scaled scores, *M* = 10, *SD* = 3

**Table 4:** Processing Speed Outcomes

Participant #	FSIQ	PSI	Symbol Search*	Coding*
1	70			
2	79			
3				
4	83	83	6	8
5	45	45	1	1
6	106	126	16	13
7	51	50	1	1
Mean	72.33	76.00	6.00	5.75
SD	22.36	37.35	7.07	5.85

*Note*: PSI = Processing Speed Index on the WISC-V and the WAIS-IV

*Scores for individual subtests are reported as scaled scores, *M* = 10, *SD* = 3

**Table 5:** Working Memory Outcomes

Participant #	FSIQ	WMI	Digit Span*	Picture Span*	Arithmetic*	Picture Memory*	Zoo Locations*
1	70						
2	79	87				10	6
3							
4	83	69	3	6			
5	45	45	1	1			
6	106	91	7	10			
7	51	55	2		2		
M	72.33	69.40	3.25	5.67			
SD	22.36	19.87	2.63	4.51			

*Note*: WMI = Working Memory Index, DS = Digit Span subtest from the WISC-V and WAIS-IV, PS = Picture Span subtest from the WISC-V, Ar = Arithmetic subtest from the WAIS-IV, PM = Picture Memory subtest fro the WPPSI-IV, ZL = Zoo Locations subtest from the WPPSI-IV

*Scores for individual subtests are reported as scaled scores, *M* = 10, *SD* = 3

**Table 6:** Executive Function Outcomes from the BRIEF and BRIEF-P*

Participant #	Inh	Shi	Em	BRI	Ini	WM	P/O	Or	Mo	Me	GEC	ISC	Fle	EM
1														
2	37	43	41			43	48				40	38	41	44
3	72	40	66			81	64				71	72	55	76
4	45	63	49	51	74	65	74	40	63	66	61			
5	45	74	67	63	68	79	61	37	49	61	62			
6	42	40	45	40	46	35	39	40	41	38	39			
7														
M	48.20	52.00	53.60	51.33	62.67	60.60	57.20	39.00	51.00	55.00	54.60	55.00	48.00	60.00
SD	13.70	15.60	12.12	11.50	14.74	20.85	13.77	1.73	11.14	14.93	14.33	24.04	9.90	22.63

Note: Inh = Inhibition, Shi = Shifting, Em = Emotional Control, BRI = Behavioral Regulation Index, Ini = Initiate, WM = Working Memory, P/O = Planning and Organization, Or = Organization of Materials, Mo = Monitor, Me = Metacognition Index, GEC = Global Executive Composite, ISC = Inhibitory Self Control, Fle = Flexibility, EM = Emergent Metacognition

*BRIEF and BRIEF-P scores are reported as T-scores (*M* = 50, *SD* = 10)

**Table 7:** Behavioral Outcomes from the BASC-2*

Participant #	Hyp	Agg	Con	Ext	Anx	Dep	Som	Int	Aty	With	Att	BSI	Adap	Soc	Lead	ADL	Func	Ada
1																		
2	32	33		31	39	38	68	48	38	46	29	31	72	45		65	46	59
3	80	53		68	43	70	63	61	82	56	70	75	58	34		40	36	40
4	37	37	49	40	44	51	36	42	46	76	69	53	38	34	30	43	28	32
5	54	45	46	48	39	53	64	53	75	81	63	65	36	31	35	24	20	26
6	40	40	43	40	32	41	35	32	41	58	36	41	57	42	48	61	55	53
7	48	41	46	45	46	58	56	54	42	47	39	45	58	61	48	60	62	60
M	48.50	41.50	46.00	45.33	40.50	51.83	53.67	48.33	54.00	60.67	51.00	51.67	53.17	41.17	40.25	48.83	41.17	45.00
SD	17.32	6.92	2.45	12.52	5.01	11.65	14.60	10.21	19.28	14.69	18.34	16.18	13.72	11.09	9.18	15.89	16.10	14.42

*Note*: Hyp = Hyperactivity, Agg = Aggression, Con = Conduct Problems, Ext = Externalizing Problems Composite, Anx = Anxiety, Dep = Depression, Som = Somatization, Int = Internalizing Problems Composite, Aty = Atypicality, With = Withdrawal, Att = Attention Problems, BSI = Behavior Symptoms Index, Adap = Adaptability, Soc = Social Skills, Lead = Leadership, ADL = Activities of Daily Living, Func = Functional Communication, Ada = Adaptive Skills

* All subscales and composite scores are reported as T-scores, *M* = 50, *SD* = 10

**Table 8:** Adaptive Behavior Outcomes from the ABAS-3

Participant #	GAC	ConC	SocC	PracC	Com*	Commu*	Func*	Hom*	Hea*	Lei*	SC*	SD*	Soc*
1													
2	95	76	116	97	1	10	3	14	6	10	9	14	17
3	72	60	87	75	2	6	1	5	6	8	5	6	8
4	78	78	82	79	6	5	4	5	7	6	9	8	7
5	51	50	58	57	1	4	1	3	2	1	2	2	3
6	88	89	83	92	9	8	8	10	9	7	9	9	7
7	66	73	74	63	6	3	5	6	2	4	5	4	5
M	75.00	71.00	83.33	77.17	4.17	5.83	3.50	7.17	5.33	6.00	6.50	7.17	7.83
SD	15.77	13.89	19.03	15.68	3.31	2.48	2.35	4.07	2.80	3.16	2.95	4.22	4.83

*Note*: GAC = General Adaptive Composite, ConC = Conceptual Composite, SocC = Social Composite, PracC = Practical Composite, Com = Communication, Commu = Community Use, Func = Functional Academics, Hom = Home Living, Hea = Health and Safety, Lei = Leisure, SC = Self-Care, SD = Self-Direction, Soc = Social

* All subscales and composite scores are reported as T-scores, *M* = 50, *SD* = 10

### Verbal abilities

Verbal abilities, reported in Table 2, were generally consistent with IQ scores across our sample. The average verbal composite score (available for six participants) fell in the borderline impaired range (*M* = 78.83, *SD* = 19.58).

### Non-verbal/perceptual abilities

There was wide variability across tests of non-verbal skill and perceptual ability, with results reported in Table 3. Nonverbal/perceptual abilities ranged from borderline impaired to low average.

### Processing speed

Processing speed scores (available for four participants), reported in Table 4, were consistent with full scale IQ, on average, and fell in the borderline impaired range (*M* = 76.0, *SD* = 37.35).

### Executive functions and working memory

On average, working memory performance (available for five participants), reported in Table 5, fell in the impaired range (*M =* 69.4, *SD* = 19.87). Comparisons between the working memory, verbal, and non-verbal/perceptual reasoning indices were made for four participants, which demonstrated reduced working memory relative to other skills (in these four patients, working memory composite = 73.0, verbal composite = 83.25, and non-verbal composite = 82.0). Caregiver ratings of executive functions from the BRIEF (available for five patients), reported in Table 6, demonstrated scores within the average range (T-scores for the Global Executive Composite; *M =* 54.6, *SD* = 14.32); however, at-risk levels of working memory difficulties were noted.

### Psychosocial outcomes

On average, caregiver ratings of psychosocial outcomes fell within the average range; however, at-risk levels of withdrawal were noted. These results are reported in Table 7.

### Adaptive abilities

On average, caregiver ratings of adaptive skills were consistent with full-scale IQ scores (*M* = 75.0, *SD* = 15.77). These results are reported in Table 8. The Social Composite was higher than the Conceptual and Practical Composites. Within specific subscales, there were relative weaknesses on functional academics and communication and relative strengths in social abilities.

## Discussion

### Neurocognitive outcomes in CCHS

On average, intellectual functioning was estimated to fall in the borderline impaired range in our sample. This is somewhat lower than prior studies, which estimated IQ to fall within the borderline to low average range [11–15]; however, the wide variability in IQ that we found in our sample is consistent with previous work. Discrepancies in average IQ between our sample and previous work with CCHS patients may be related to small samples sizes across studies.

Working memory emerged as a relative weakness in our sample. This is important and should be explored further in future studies, as working memory is a crucial cognitive process for learning, including reading [24] and math [25]. Moreover, while working memory is considered a component of executive function in many theoretical models [26], we did not employ other executive function tests in this study. It is important to evaluate whether this is a general area of weakness in this population or if there may be a deficit specific for the holding and processing of material in working memory.

### Psychosocial outcomes in CCHS

Coping skills, symptoms of emotional difficulties (i.e., anxiety, depression), and behavioral difficulties (i.e., attention problems, impulsivity, etc.) fell within the average range in this sample. This is consistent with findings from Marcus et al. [12] and suggests that psychological wellbeing may be a promising protective factor that can be leveraged in this population. This is also consistent with a study from Ruof et al. [14], which reported behavioral functioning generally falling in the average range despite impairments in intellectual and adaptive functioning. The only at-risk area was the withdrawal subscale, which evaluates the extent to which the individual may avoid others and keep to himself or herself. However, it is possible that this finding was due to circumstances that are secondary to having a complex medical condition, including hospitalizations, numerous doctors appointments, and missing days of school. Overall, findings in this domain are favorable for this population and suggest that individuals with CCHS are resilient and able to cope with their disease effectively.

### Adaptive outcomes in CCHS

Adaptive outcomes were consistent with IQ scores in this sample, which was expected since these abilities tend to covary with one another, particularly among individuals with delayed or disordered development [27]. This is consistent with the only prior study that assessed adaptive skills in this population [14]. A relative weakness was noted in communication skills; however, this is likely secondary to ventilatory dependence and need for tracheostomy [28]. Social skills emerged as a relative strength, despite a weakness in communication. Because well-developed social skills are associated with behavioral and adaptive skill development, as well as mental health outcomes [29], we believe this is another protective factor that may help bolster outcomes in this population.

### Implications for individuals with CCHS

Findings linking neurocognitive functioning to PARMs have important implications for early identification and treatment of individuals with CCHS. For instance, children with the 20/26 and 20/27 genotypes would likely benefit from early intensive interventions to bolster later cognitive abilities. However, because neurocognitive testing often occurs when the child is a toddler or early school aged, genetic testing can precede this evaluation and provide some insight into the child’s level of risk. Although genetic information can help guide medical care for CCHS patients, our findings support the recommendation from the American Thoracic Society [1] that all individuals with CCHS should receive a comprehensive neuropsychological evaluation to document cognitive strengths and weaknesses in order to inform diagnostic and treatment recommendations.

### Limitations and future directions

Our conclusions must be considered in the context of a few limitations, including our small sample. We were unable to perform sophisticated statistical analyses because of our small sample and lack of consistency in tests across participants. It will continue to be difficult for research groups to obtain larger samples of individuals with CCHS. Therefore, it is our recommendation that research groups with access to this population collaborate by utilizing a similar testing battery, compiling databases across labs, and conducting more rigorous statistical analyses with this population.

More in-depth cognitive testing, particularly within the domains of working memory and other executive functions, will be important for future research in order to better understand the specific deficits that are common in this population. Future studies should also consider including academic screening tests (reading and math).

With regard to genetics, more work is needed to further understand the heritability of CCHS. It may be helpful to evaluate cases in which multiple family members across multiple generations have been diagnosed.

Despite these limitations, we believe we have contributed important knowledge to the field’s understanding of CCHS because we are the first to integrate neurocognition, psychosocial skills, adaptive abilities, genetics, and need for ventilatory support.

## Conclusions

Our findings support the need for comprehensive neuropsychological evaluation in individuals with CCHS. Genetic testing in infancy should precede neuropsychological testing and may be used to provide preliminary prognostic information about the child’s risk status. Our findings demonstrated a relative weakness in working memory, which should be considered in future studies. Additionally, findings from psychosocial and adaptive evaluation highlight a number of protective factors in this population, including good coping skills and relatively strong social skills.

## Data Availability

The datasets used and/or analyzed during the current study are available from the corresponding author on reasonable request.

## Abbreviations

ABAS-3: Adaptive Behavior Assessment Scales, Third Edition
ANS: Autonomic nervous system
BASC-2: Behavioral Assessment System for Children, Second Edition
Bayley-III: Bayley Scales of Infant and Toddler Development, Third Edition
BRIEF: Behavioral Rating Inventory of Executive Function
CCHS: Congenital central hypoventilation syndrome
CPAP: continuous positive airway pressure
IQ: intelligence quotient
M: mean
NPARMS: non-polyalanine repeat expansion mutations
PARMS: polyalanine repeat expansion mutations
PHOX2B: Paired-like homeobox 2B
SD: standard deviation
WAIS-IV: Wechsler Adult Intelligence Scale, Fourth Edition
WISC-V: Wechsler Intelligence Scale for Children, Fifth Edition
WPPSI-IV: Wechsler Preschool and Primary Scale of Intelligence, Fourth Edition
